# Optical Ammonia Sensors Based on Spray-Coated Polyaniline Complexes with Polysulfonic Acids

**DOI:** 10.3390/s25113348

**Published:** 2025-05-26

**Authors:** O. L. Gribkova, V. A. Kabanova, E. I. Rodina, M. A. Teplonogova, L. I. Demina, A. A. Nekrasov

**Affiliations:** 1 Frumkin Institute of Physical Chemistry and Electrochemistry of the Russian Academy of Sciences, Leninskii prospect 31, 119071 Moscow, Russia; 2 Kurnakov Institute of General and Inorganic Chemistry of the Russian Academy of Sciences, Leninskii prospect 31, 119071 Moscow, Russia

**Keywords:** polyaniline complexes, polyacid, spray coating, ammonia sensors, optical gas sensors

## Abstract

The optical ammonia-sensing properties of water-dispersible polyaniline (PANI) complexes chemically synthesized in the presence of polysulfonic acids of different structure and chain flexibility were compared for the first time. Flexible-chain poly(styrene-4-sulfonic acid) and poly-(2-acrylamido-2-methyl-1-propanesulfonic acid), as well as semi-rigid-chain poly-4,4′-(2,2′-disulfonic acid)diphenylene-iso-phthalamide and rigid-chain poly-4,4′-(2,2′-disulfonic acid)diphenylene-tere-phthalamide (t-PASA) were used. The sensor films were prepared by a convenient and scalable method—spray coating of aqueous solutions on glass substrates. The optical response time and amplitude of the sensor films in the range of ammonia concentrations from 5 to 200 ppm were investigated. To overcome the influence of humidity and presence of over-stoichiometric protons of the polyacid on the accuracy of ammonia determination treatments of the films in aqueous solutions of NaCl, CaCl_2_ and BaCl_2_ were tested. The treatment in 1 M CaCl_2_ solution for all of the PANI complexes results in a significant improvement in the response time, amplitude and reproducibility. The films of PANI complexes with the flexible-chain polyacids have the highest response amplitude in the range of ammonia concentrations 5–25 ppm. PANI-t-PASA film demonstrated the best sensory properties at ammonia concentrations more than 50 ppm. FTIR spectroscopy showed that CaCl_2_ treatment results in cross-linking of sulfoacid groups from adjacent polyacid chains by Ca^2+^ ions. Thus, such a treatment results both in the neutralization of excessive protons and a significant reduction in the films’ swelling at high humidity.

## 1. Introduction

One of the current trends in the progress of materials science is the development and production of new functional materials with predetermined properties. From this point of view, electrically conductive polymers (ECP) are of great interest. The advantages of these polymers (polyaniline (PANI), poly(3,4-ethylenedioxythiophene) (PEDOT) and polypyrrole (PPy)) show high conductivity and optical transparency in the conducting state, and high stability in the doped state. They have a unique combination of physicochemical, electrical and optical characteristics that makes these materials promising for use in chemical sensors [1,2,3].

PANI is distinguished by the capability of detecting both oxidation-reduction and pH active gases, such as ammonia, amines, etc. [4]. Most of these gases are dangerous to humans and are among the most common industrial pollutants. Also, the detection of amines is one of the methods for controlling freshness, quality and safety of food products [5].

Chemical sensors can be classified based on their operating principle: electrochemical [6], thermochemical [7], resistive [8,9,10], mass-sensitive [11] and optical [12]. The majority of ammonia and amines sensors based on conductive polymers operate using the resistive detection mechanism [1,2]. For example, PANI obtained by chemical synthesis in the presence of hydrochloric acid and indium oxide nanotubes was used for detecting sprayed pesticides [13]. The resistive method of ammonia determination is simple to implement and is currently well studied. However, the resistance value can be affected by external factors. At the same time, optical sensors can provide high sensitivity and short response time, while they are less affected by external factors such as electromagnetic interference, humidity and temperature. Also, the optical analytical signal can be transmitted over long distances without distortion [1,14].

Upon exposition to ammonia, PANI film undergoes deprotonation, which results in a transition from the salt form to the base one [15]. This change in the PANI electronic structure is reflected in the change in the absorption spectrum of the film.

PANI films can be obtained in various ways, for example, by electrochemical deposition on a conductive substrate during electropolymerization. Chemically synthesized PANI can be applied on a substrate using various methods: casting onto a horizontal substrate, spin coating, spray coating, inkjet printing, etc. Many studies have shown that the method of film fabrication significantly affects both the mechanical properties and morphology of the resulting film (adhesion to the substrate, thickness uniformity, roughness), as well as the sensory properties [12,15,16].

The comparative studies of the optical ammonia-sensing properties of PANI, PEDOT and polypyrrole obtained electrochemically in inorganic electrolytes [17] and in the presence of polyelectrolytes of different structure [12] were presented, and the prospects of such films for ammonia detection in air were shown. Also, double layer films of PANI/PEDOT electrodeposited in inorganic electrolytes were used as optical ammonia sensors [18,19].

The literature contains enough work on the development of optical sensors for ammonia based on chemically synthesized PANI. PANI films obtained in the presence of dodecylbenzenesulfonic and laurylsulfonic acids and TiO_2_ nanoparticles can be used to detect ammonia in solution [20]. A significant influence of the film deposition method (sedimentation from the synthesis solution or spin coating) and the nature of dopant (hydrochloric acid, camphorsulfonic acid or iodine) on the sensitivity of PANI to ammonia was demonstrated [15]. A more developed surface area of the film contributes to the improvement of sensing characteristics. An optical sensor for detecting ammonia and amines that can be connected to a smartphone was developed [5]. It is intended to be used to determine the freshness of fish. It was shown that a chemically synthesized complex of PANI with polystyrene sulfonic acid can be applied to filter paper and used as a colorimetric sensor for amines [21].

The synthesis of PANI in the presence of polysulfonic acids of various structures makes it possible to control the optical and electrical properties of the resulting PANI complexes, the morphology of their films [22,23] and, consequently, their sensory characteristics. The use of water-soluble polysulfonic acids in PANI synthesis allows the preparation of water-soluble polymer compositions, films of which can be applied to various substrates, including flexible ones, using scalable methods (spray coating, inkjet printing, etc.).

The deposition of PANI layers by inkjet printing has been mostly used to create biological and chemical sensors based on the resistive sensing mechanism [24,25]. In this case, solutions of PANI synthesized in the presence of camphorsulfonic, dodecylbenzenesulfonic and polystyrene sulfonic acids were used for printing. High-boiling organic solvents (dimethyl sulfoxide, N-methylpyrrolidone) or water with the addition of sodium dodecyl sulfonate served as solvents in these works. Resistive sensing properties of films prepared by spray coating and inkjet printing of aqueous solutions of PANI synthesized chemically in the presence of polyvinylpyrrolidone were compared in [16]. The studies showed that the spray-coated film has 2–3 times greater roughness and demonstrates twice the conductivity change. PANI obtained in the presence of dodecylbenzenesulfonic acid in water can be used to determine the level of ammonia in the blood by spectrophotometry or electrochemical impedance spectroscopy [6]. A composition of PANI with CuCl_2_ applied by spray coating onto paper was used as a hydrogen sulfide sensor [26]. Also, PANI compositions with 2-D carbides of transition metals deposited on cotton fabrics were developed for detection of ammonia [14]. Most of the above mentioned PANI compositions were prepared based on conventional water-insoluble PANI prepared in HCl, which was then processed and/or mixed with various additives to obtain water-dispersible PANI compositions. Only in the work [24], aqueous solutions of PANI were prepared in the presence of an organic sulfonic acid for designing printed flexible humidity sensors and in [27] a composite of cellulose/PANI–poly-(2-acrylamido-2-methyl-1-propanesulfonic acid) was used as resistive humidity sensor and biomedical sensor for heart rate or respiration activity. Spray-coated films of PANI complexes with sulfonated polysulfone demonstrated good sensing properties in optical sensors for ammonia in air [28].

Earlier, we proposed treating the films of PANI complexes with 1M aqueous solution of CaCl_2_. Such treatment did not influence the electronic structure or morphology of PANI films and was shown to decrease the solubility of drop-cast films of chemically synthesized PANI complexes with polyacids [22]. Such treatment has also led to an improvement in the ammonia-sensing properties of electrochemically deposited films of ECP complexes with sulfonated polyelectrolytes [12,29].

A more or less similar approach was used in [30]: the treatment of inkjet-printed PEDOT:PSS film on paper in FeCl_3_ solution improved the ammonia-sensing properties. The authors hypothesized that the sensor’s high humidity tolerance was achieved by the molecular interaction between the sulfonate groups in PSS and the iron(III) ions which suppressed the swelling of the PSS shell surrounding PEDOT in high humidity, resulting in the maintenance of electronic coupling between PEDOT chains. Also, calcium salts are commonly used for crosslinking of biopolymers such as alginate [31,32] and pectin [33] to prepare hydrogels with the purpose of improving their mechanical properties (including swelling) and/or regulate their gas and vapor permeability.

In this work, we have first performed a comparative study of the optical ammonia-sensing properties of water-dispersible PANI complexes chemically synthesized in the presence of polysulfonic acids of different structures and chain flexibility. The films were prepared by spray coating onto transparent glass substrates. The effect of the structure and flexibility of the polyacid dopants on the morphology spray-coated films and ammonia-sensing properties is considered. Also, the influence of treatment of PANI films in aqueous solutions of different cations (Na^+^, Ca^2+^, Ba^2+^) on the ammonia-sensing properties is discussed.

## 2. Materials and Methods

### 2.1. Materials

**Figure 1:**
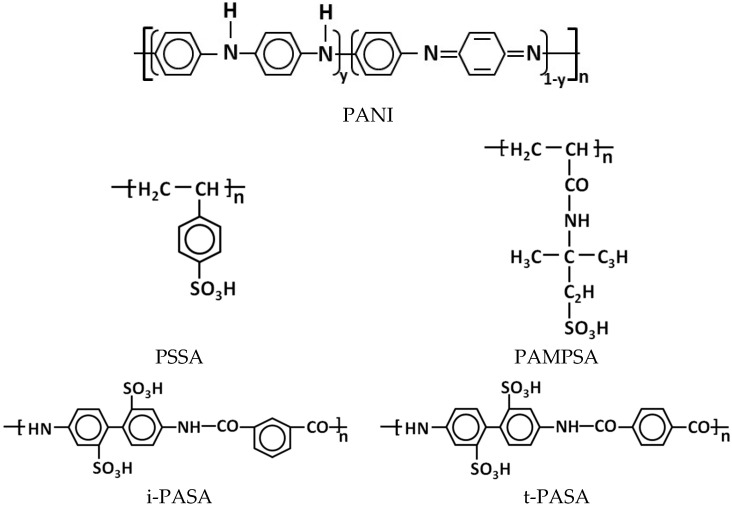
Chemical structure of PANI and polysulfonic acids used in the paper.

PANI was synthesized by oxidative chemical polymerization of aniline in aqueous solutions of polymeric sulfonic acids of various structures (Figure 1): flexible-chain poly(styrene-4-sulfonic acid) (PSSA) and poly-(2-acrylamido-2-methyl-1-propanesulfonic acid) (PAMPSA), as well as semi-rigid-chain poly-4,4′-(2,2′-disulfonic acid)diphenylene-iso-phthalamide (i-PASA) and rigid-chain poly-4,4′-(2,2′-disulfonic acid)diphenylene-tere-phthalamide (t-PASA). The polymerization was carried out at room temperature (~24 °C) according to the method described in [22,34]. Prior to the synthesis, aniline (Sigma-Aldrich, St. Louis, MO, USA, reagent grade) was distilled under reduced pressure with nitrogen bubbling. PAMPSA (MW 2,000,000, 15% aqueous solution) was purchased from Sigma-Aldrich. Sodium salts of i-PASA, t-PASA were synthesized as described in [34,35]. PSSNa (Sigma-Aldrich, MW 1,000,000, 25% aqueous solution), i-PASNa and t-PASNa were converted into H^+^-forms using ion-exchange column. All polyacid solutions were purified via dialysis (cellulose membrane ZelluTrans MWCO 8000–10,000, Roth, Karlsruhe, Germany) against 18 MOhm deionized water for 3 days and diluted by 18 MOhm deionized water to achieve necessary concentration. We have determined viscosity-average molar weight of polysulfonic acids using the method described in [34]: PSSA—600,000, PAMPSA—1,100,000, i-PASA—17,000, t-PASA—59,000.

The ratio of the concentrations of aniline to sulfoacid groups of the polyacids was 0.5 mol/g-eq. of sulfogroups: for the single base polyacids (PAMPSA, PSSA) one aniline molecule corresponded to two monomer units of the polyacid, while for the double base polyacids (t-PASA and i-PASA) (Figure 1) this ratio was 1:1. Ammonium persulfate (APS, Sigma-Aldrich, reagent grade) was used as the oxidizing agent, and the ratio of the concentrations of aniline to APS was 1:1 mol/mol. The concentration of aniline was 0.01 M for the synthesis in the presence of PAMPSA (0.02 g-eq. SO_3_), PSSA (0.02 g-eq. SO_3_) and i-PASA (0.01 g-eq. SO_3_). In the case of the synthesis in t-PASA, in order to prevent sedimentation of the resulting solution and ensure it is suitable for spray coating, the aniline and the polyacid were taken in 0.005 M and 0.005 g-eq. SO_3_ concentrations, respectively. The progress of the synthesis was controlled by in situ registration of electronic absorption spectra (AvaSpec 2048 spectrophotometer, Avantes BV, Apeldoorn, The Netherlands) of the reaction solution. After the polymerization was completed, the PANI complexes with polyacids were purified from oligomers, residues of unreacted oxidizer and aniline via dialysis (ZelluTrans MWCO 8000–10,000) against deionized water for 3 days. The mass concentrations of the aqueous solutions of PANI complexes thus obtained were: 3.7 g/L PANI-PSSA, 4.9 g/L PANI-PAMPSA, 3.2 g/L PANI-i-PASA, 2.9 g/L PANI-t-PASA.

The films of PANI complexes were obtained by spraying the aqueous solutions onto glass substrates. Before spraying, the solutions were treated in an ultrasonic bath for 10 min. The substrates were placed onto a horizontally leveled platform of an IKA MSC BASICS magnetic stirrer (IKA-Werke GmbH, Staufen, Germany), heated up to 70–80 °C. After thermal stabilization of the glass substrates during 3–4 min, spray coating was performed using a JAS 1147 aerograph (nozzle diameter 0.3 mm, JAS, Shanghai, China), fixed at a distance of 20 cm from the platform. The solutions were spray-coated step by step, with drying intermediate layers.

Samples for investigating the sensing properties were prepared as follows: for each PANI–polyacid complex, 5 samples were taken as-coated, 5 samples were treated during 30 min in 1 M aqueous solution of NaCl, 5 samples in CaCl_2_ and 5 samples in BaCl_2_. After the treatment excess of the salts’ solutions was removed from the films by keeping them in deionized water for 5 min, followed by air drying (Figure S1, Supplementary materials, see the link after the Conclusions).

### 2.2. Characterization Techniques

The thickness of the films was measured by the MII-4 microinterferometer (LOMO, St. Petersburg, Russia). The thicknesses of PANI–polyacid films depending on the polyacid used were (nm) as follows: 275 ± 125 (PANI-PSSA), 375 ± 50 (PANI-PAMPSA), 185 ± 15 (PANI-i-PASA) and 425 ± 25 (PANI-t-PASA). The wide spread of thickness for PANI-PSSA is due to its rough surface (see AFM data below), which increases the possibility of error in thickness determination by interferometry. The differences in thickness are explained by (1) different molecular weights of the polyacids; (2) possibly different yield of aniline polymerization in the presence of different polyacids; (3) necessity to prepare films with comparable absorbance changes in the spectral area of optical sensor response to ensure accuracy of the determination.

The surface morphology of PANI films was recorded using Enviroscope atomic force microscope (AFM) with a Nanoscope V controller (Bruker, Billerica, MA, USA) in tapping mode. The roughness of PANI films was averaged from 5 different areas of 3 films. Scanning electron microscopy (SEM) images were taken using a Tescan Amber GMH scanning electron microscope. Images were obtained using Everhart-Thornley SE detector at ×3000–100,000 magnifications and at an accelerating voltage of 0.5–1.0 kV.

The ammonia-sensing properties of PANI films were studied similarly as described in [29]. The spray-coated films on glass substrates were placed into a closed 5 cm spectrophotometric quartz cell filled with ammonia vapors in equilibrium (at 22–25 °C), with a 5 mm layer of the aqueous solutions of different ammonia concentrations on the bottom of the cell (Figure S2). Using literature reference data on the equilibrium concentrations of ammonia in the aqueous and gas phases at 25 ± 0.1 °C [36], we have calculated the volume of the 30% NH_3_ solution (analytical grade, Chimmed, Russia) necessary to obtain different NH_3_ concentrations in 3 mL of water. Then, the concentrations of ammonia in water were recalculated to the concentrations in air expressed in ppm.

UV–visible–NIR (350–1000 nm) absorption spectra of PANI films in air and their evolution when exposited to the ammonia vapors was registered in situ using the AvaSpec 2048 spectrophotometer. The recording time of each spectrum was 2 s.

The sensor response (ΔA) was calculated as the relative variation of the absorbance amplitude at characteristic wavelengths (different for different PANI complexes)

(1) $$∆A=\frac{A_{{NH}_{3}vap}-A_{air}}{A_{air}}100\%$$

where A_NH3vap_ is the value of absorbance when the sample is exposed to NH_3_, and A_air_ is the value of absorbance when the sample is exposed to air.

The response time (t_r_) was calculated as the time necessary to reach 90% of the response amplitude. The diffusion coefficient (D) was calculated as described in [12]

(2) $$\frac{A_{t}-A_{0}}{A_{k}-A_{0}}=\frac{2}{l}{\left(\frac{D_{t}}{\pi }\right)}^{0.5}$$

where A_0_ and A_k_ are the optical absorbance at characteristic wavelength in the initial and final moments of sensing, respectively, A_t_ is the optical absorbance at characteristic wavelength at the time t and l is the film thickness.

FTIR spectra were registered in the range from 4000 to 400 cm^–1^ on a Nicolet NEXUS scanning single-beam Fourier transform IR spectrometer (CsI beam splitter, TGS–CsI detector, photometric accuracy of 0.1%, resolution of 2 cm^–1^). The samples were spray-coated on pieces of Ge wafer. The spectra were recorded in transmission mode. The measurements were performed under standard conditions.

## 3. Results and Discussion

### 3.1. Spectral Changes in PANI Films During Exposition to Ammonia

**Figure 2:**
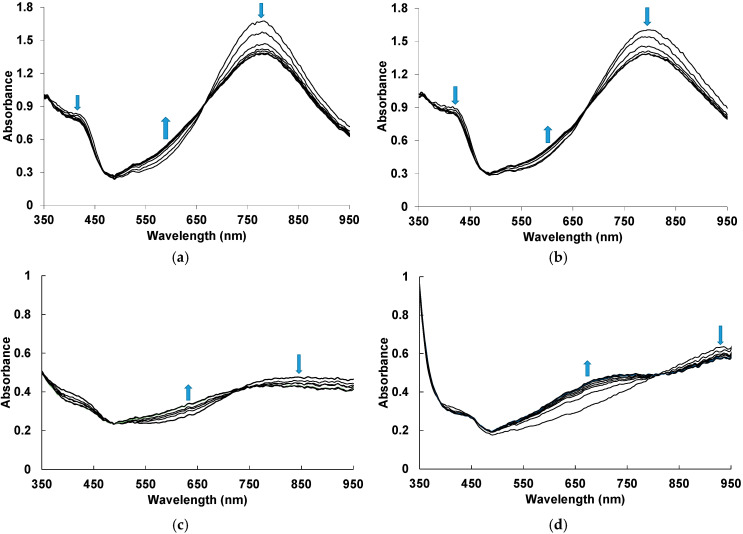
Time evolution of UV–visible–NIR electronic absorption spectra of PANI-PSSA (**a**), PANI-PAMPSA (**b**), PANI-i-PASA (**c**) and PANI-t-PASA (**d**) films during exposition to 50 ppm NH_3_. The blue arrows indicate simultaneous growth/drop of absorbance at characteristic wavelengths.

Typical evolutions in time of the electronic absorption spectra of the films of PANI complexes with different polyacids measured in situ during exposition to 50 ppm NH_3_ are presented in Figure 2. The blue arrows indicate simultaneous growth/drop of absorbance at characteristic wavelengths. One can see that PANI complexes have different electronic structures depending on the structure of polyacid. The flexibility of the polyacid chain, the distance between the sulfoacid groups on the polymer chain and the length and rigidity of the side chains containing sulfoacid groups affect the character of the synthesis and the spectral properties of the PANI complexes [22]. The presence of rigid-chain polyacids (Figure 2c,d) in the complexes facilitates the electron exchange between neighboring PANI chains, which is manifested in the increased absorption in the NIR spectral range. In contrast, flexible-chain polyacids (Figure 2a,b) are able to adjust their conformation to the conformation of PANI and possibly isolate PANI chains from each other, which leads to increased absorption of localized polarons (800 nm) [37].

In the case of PANI complexes with flexible-chain PSSA and PAMPSA (Figure 2a,b), during exposition to ammonia we observe the decrease in absorption in the region of localized polarons and near 420 nm, which is related to the radical cations [37]. Simultaneously, an increase in absorption in the region of 500–650 nm corresponding to the deprotonated form of PANI [37,38] is observed. Such changes indicate the transition of PANI from the salt to the base form. Two isosbestic points confirm mutual transitions between the fragments of chemical structure of PANI. For PANI complexes with rigid-chain i-PASA and t-PASA (Figure 2c,d), one can see the noticeable absorption growth in the range of 600–750 nm.

For building the optical response transients, we have chosen the wavelength areas where response amplitudes were highest: for PANI-PSSA and PANI-PAMPSA—570 nm, for PANI-i-PASA—630 nm and for PANI-t-PASA—670 nm.

### 3.2. Specific Features of Ammonia-Sensing Properties of PANI–Polyacids Films

When studying the sensing properties of PANI complexes with polyacids, some specific features should be taken into account. First, hydrogen ions in the films of PANI complexes with polyacids can easily migrate through sulfonic acid centers to the film surface, where they can react with ammonia molecules. This can lead to a faster sensor response. The second important feature is possible neutralization of some ammonia molecules by excessive protons of the polyacids, which are not ionically linked with the positively charged fragments of PANI chain. This neutralization does not cause spectral changes in the PANI films, thus reducing the response amplitude. The over-stoichiometry excess of sulfoacid groups is needed to ensure reproducible chemical synthesis of PANI–polyacid complexes [34]. However, the presence of these excessive protons can reduce the sensitivity of the films.

To solve the latter problem, we have tried treatment of the films of PANI–polyacid complexes by aqueous solutions of chlorides of various metals (Na^+^, Ca^2+^, Ba^2+^) and compared the influence of such treatment on the ammonia-sensing properties. The aim of using NaCl-treatment was to replace excessive protons of polyacids by cation exchange process. Ca^2+^, Ba^2+^ were chosen because they can form strong ionic bonds with sulfonic groups of polyacids by analogy with weakly soluble CaSO_4_ and BaSO_4_. In addition to the replacement of excessive protons Ca^2+^, Ba^2+^ decrease solubility of PANI complexes by creating bridges between sulfonic groups belonging to adjacent polyacid molecules (or different fragments of flexible polyacid chain).

Unfortunately, the treatment of all PANI complexes with NaCl results in unstable sensory properties (a large spread of response amplitudes, shown in Figure S3a,b), which may be due to the influence of humidity. In our opinion, humidity may cause the swelling of PANI films, as increasing their thickness resulted in a change in the balance of the absorption/refraction/reflection phenomena at the air/film/glass interfaces. Since the polymer film has submicron thickness, this influences optical properties of the films in the visible range of spectrum. Treatment with Ba^2+^ ions gives a slightly lower response amplitude (Figure S3e,f) compared to that in the case of Ca^2+^ treatment (Figure S3c,d). The reason for this may be the significantly lower solubility of Ba-SO_3_ salt, resulting in crosslinking and neutralization of the sulfoacid groups only on the surface of the film, thus hindering further penetration of the neutralizing agent to the bulk. Therefore, further investigations in this work were performed for CaCl_2_ treatment only.

**Figure 3:**
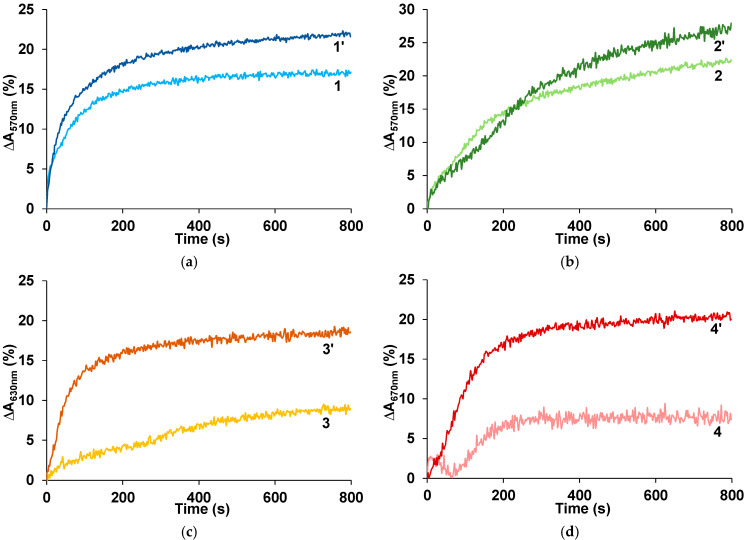
Response transients at the exposition to 25 ppm of ammonia for the films of PANI complexes with PSSA (**a**), PAMPSA (**b**), i-PASA (**c**) and t-PASA (**d**), untreated (1, 2, 3, 4) and treated with CaCl_2_ (1′, 2′, 3′, 4′).

According to the World Health Organization, workplace concentration limit of ammonia in the air at 8 h exposure is 25 ppm [39]. Figure 3 shows response transients at the exposition to ammonia concentration of 25 ppm for the PANI films untreated and treated by Ca^2+^ ions. It is clearly seen in Figure 3c,d that the treatment of the PANI complexes with rigid-chain polyacids leads to a greater increase in the response amplitude than in the case of PANI complexes with flexible-chain polyacids (Figure 3a,b). At the same time, for all treated PANI complexes, the response amplitudes are comparable.

On the base of the time dependences of the optical absorbance at the chosen wavelengths, we have calculated the dependences of (A_t_ − A_0_)/(A_k_ − A_0_) on square root of time (Figure S4) in accordance with the Equation (2). From the linear parts of these dependences, we have found the values of the ammonia diffusion coefficients (Table 1). The S-shaped graph indicates the deviation of ammonia diffusion in these films from Fick’s second law. It may be due to structure relaxation (changes in the polymer structure and/or conformation upon deprotonation) accompanying the diffusion of ammonia in the PANI film. The rate of these processes may be comparable to the ammonia diffusion rate.

From Table 1, it is seen that after the treatment by CaCl_2,_ the diffusion coefficients increase for all PANI films: PANI-PAMPSA~5 times; PANI-PSSA, PANI-i-PASA~3 times; PANI-t-PASA~2 times. The response times of all films decrease by about two times. This value does not correlate with the change in diffusion coefficients due to different morphology of PANI complexes (see below). The treatment positively affected the response amplitudes: PANI-PAMPSA-growth~2 times; PANI-PSSA~2.2 times; PANI-t-PASA~2.3 times; PANI-i-PASA~2.8 times. Importantly, PANI-t-PASA exhibits the highest response amplitude, which directly influences the accuracy of ammonia detection.

FTIR spectroscopy was used to reveal possible interactions between the cations (Na^+^, Ca^2+^) and sulfoacid groups of polyacids in PANI complexes. Since PANI-PAMPSA is a composite of polyaniline and sulfonic acid, the FTIR spectrum contains bands that describe the vibrations of PANI (1608, 804 cm^−1^) and bands that describe the vibrations of exclusively PAMPSA (1652, 1040, 720, 625 cm^−1^), as well as combined bands that include both PANI and PAMPSA (3301, 3260, 3065, 2997, 2986, 2941, 2919, 2850, 1555, 1459, 1393, 1372, 1301, 1215, 1181, 1155 cm^−1^ (Figure S5)). As we see, most bands are combined.

Since the treatment of PANI-PAMPSA with NaCl and CaCl_2_ leads to deprotonation of the acid to form sulfonic acid salts, the most significant changes in the FTIR spectra are to be expected for the bands describing the sulfoacid fragment: asymmetric stretching ν_as_(O=S=O), symmetric stretching ν_s_(O=S=O) and stretching ν(S-O).

To determine the spectroscopic criteria of PAMPSA binding in the composition of PANI-PAMPSA, a preliminary experiment was carried out for PAMPSA+Na^+^ and PAMPSA+Ca^2+^ films (Figure S5). It is shown that in the PAMPSA+Na^+^ and PAMPSA+Ca^2+^ spectra, a shift of the SO_3_**^-^** anion vibration bands relative to the position of the corresponding bands in the sulfonic acid spectrum is recorded (Figure S5). These results allowed us to unambiguously define bands for the analysis of sulfogroup.

**Figure 4:**
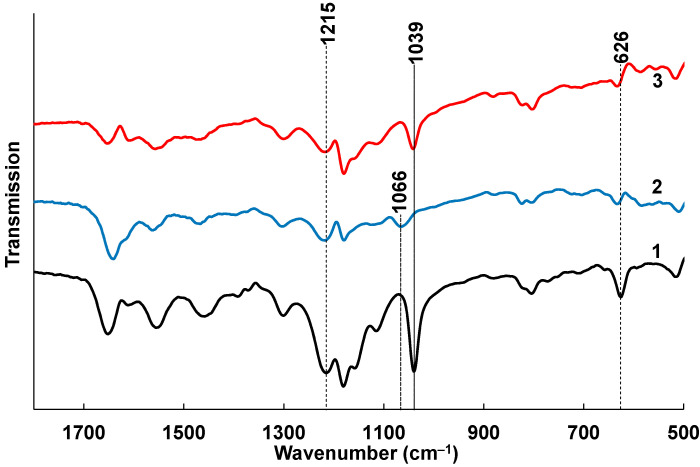
The FTIR spectra of spray-coated PANI-PAMPSA film (1), treated with CaCl_2_ (2) and NaCl (3).

In the PANI-PAMPSA spectrum (Figure 4, curve 1), the sulfonic acid group is characterized by the bands: ν_as_(O=S=O) 1215 cm^−1^, ν_s_(O=S=O) 1039 cm^−1^ and ν(S-O) 626 cm^−1^. It should be noted that the band at 1215 cm^−1^ is combined of stretching ν(C-N•+) in the polaron lattice of PANI [41] and ν_as_(O=S=O), but the main contribution to the position and intensity of this band is made by an asymmetric stretching vibration.

As observed in the preliminary experiment (Figure S5), the deprotonation of the acid with the formation of PAMPSA+Na^+^ does not lead to significant changes in the bands characterizing ν(O=S=O) (Figure 4, curve 3). A shift to the high-frequency region at 7 cm^−1^ is recorded for the ν(S-O) band, which is certainly explained by deprotonation of O_2_SOH group. However, the formation of a calcium salt linker chain in PAMPSA+Ca^2+^ leads, in addition to a similar (as for PAMPSA+Na^+^) shift of the ν(S-O) band at 7 cm^−1^, to a significant shift of the symmetric stretching band ν_s_(O=S=O) at 27 cm^−1^ to the high-frequency region (Figure 4, curve 2). The shift of this band can be used as a criterion for the formation of a salt with Ca^2+^, leading to the formation of a chain structure rather than simple deprotonation (as in the case of Na^+^).

Thus, the treatment with CaCl_2_ leads to cross-linking of the polyacid matrix by strong ionic bonds of Ca^2+^ ions with two sulfogroups belonging to neighboring polyacid chains. This leads to an effective substitution of the excessive protons of the polyacids.

### 3.3. Morphology

**Figure 5:**
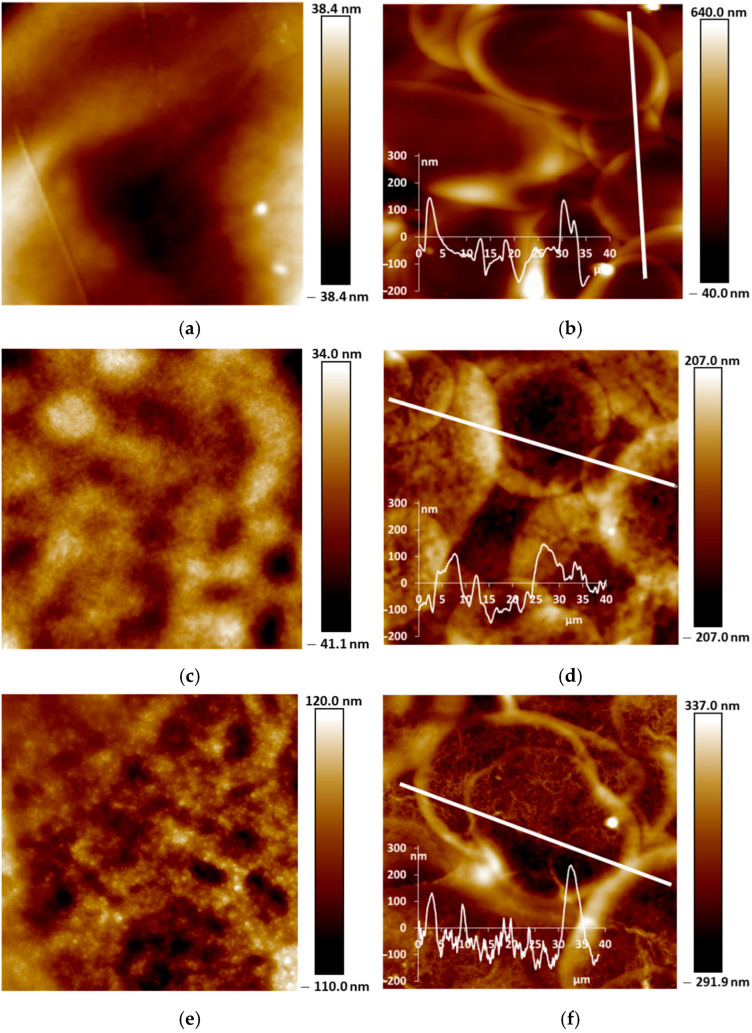

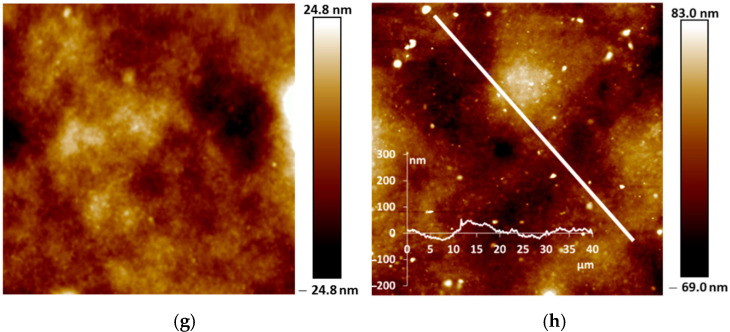
AFM images of PANI-PSSA (**a**,**b**), PANI-PAMPSA (**c**,**d**), PANI-i-PASA (**e**,**f**) and PANI-t-PASA (**g**,**h**) films applied by the spray coating method in 5 µm × 5 µm (**a,c,e,g**) and 40 µm × 40 µm (**b,d,f,h**) scales.

**Figure 6:**
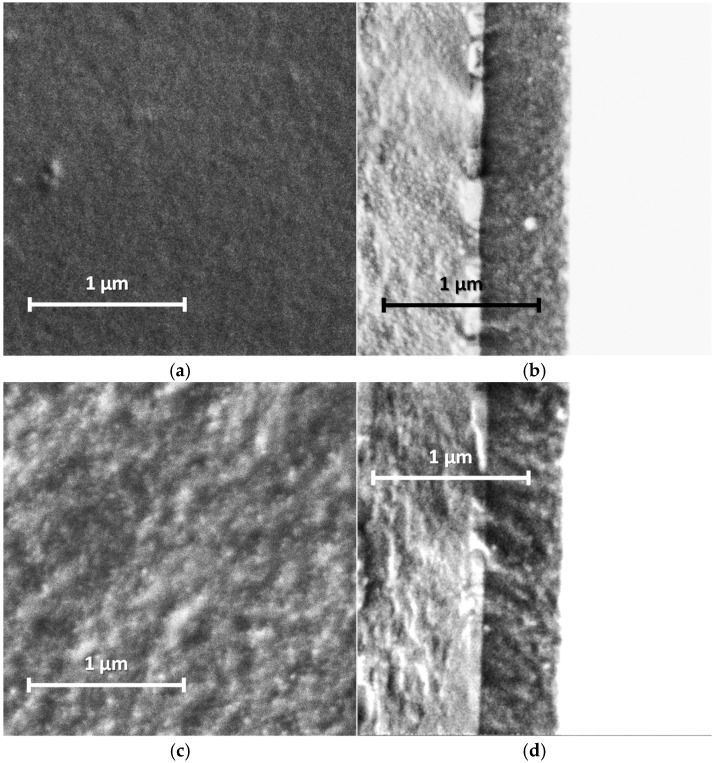

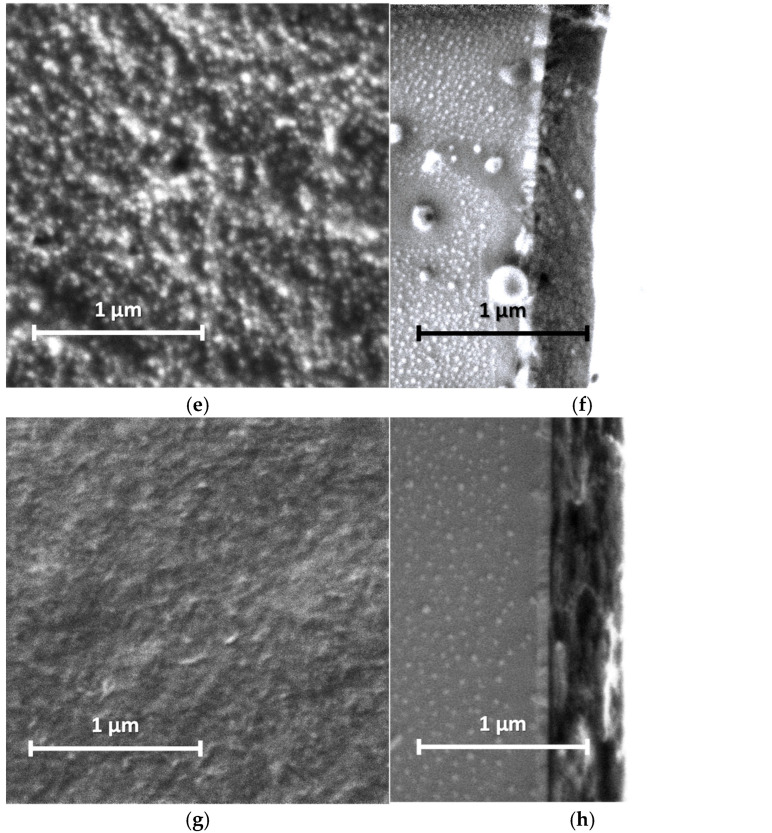
SEM images of PANI-PSSA (**a**,**b**), PANI-PAMPSA (**c**,**d**), PANI-i-PASA (**e**,**f**) and PANI-t-PASA (**g**,**h**) films applied by the spray coating method: planar (**a**,**c**,**e**,**g**) and cross-sectional (**b**,**d**,**f**,**h**) views. Cross-sections from left to right: glass, ITO, PANI.

Our studies of the morphology of spray-coated films revealed that they are more dense and uniform and have roughness lower in small scale (5 µm × 5 µm) than the electrochemically synthesized films [12]. This is due to the coating procedure, which includes drying at each step of spraying of nano-sized aqueous dispersions. AFM and SEM images of the PANI films are shown in Figure 5 and Figure 6. One can see the presence of macro-roughness with abrupt height peculiarities on the scale of 40 µm × 40 µm and the intrinsic roughness on the scale of 5 µm × 5 µm. PANI−PSSA film has a very uniform and smooth morphology on the small scale (Figure 5a and Figure 6a). At the same time, the macro-roughness of PANI-PSSA is the highest—150 nm, and oval droplets from 14 to 25 µm are clearly visible, with a height difference of up to 400 nm on the 40 µm × 40 µm scale (Figure 5b). PANI-PAMPSA film has fibrous morphology (Figure 5c and Figure 6c) and consists of round, dried-up droplets, with the diameter from 15 to 20 µm; the height of the edges of droplets is 100 nm (Figure 5d). The cross-section of PANI-PAMPSA and PANI-PSSA revealed a very dense and homogeneous structure, with lower nanorelief in the case of PANI-PSSA (Figure 6d,b).

For PANI-i-PASA, particles with a diameter of 50–60 nm are clearly visible (Figure 5e and Figure 6e), dried-up droplets, with a diameter from of 20 to 30 µm; the edges of the drops are thin—4 µm; the height of the edges is up to 200 nm (Figure 5f). One can observe an even finer internal morphology of the films with barely noticeable layers along the substrate (Figure 6f).

In the case of PANI-t-PASA, the droplets are practically not pronounced and the height fluctuations do not exceed 50 nm (Figure 5h). The roughness of PANI-t-PASA films is the smallest—22 nm. PANI-t-PASA film has uniform, fibrous morphology on a small scale (Figure 5g and Figure 6g). At the same time, on a cross-section, one can see the layered-like structure (Figure 6h). It is seen that the PANI-t-PASA layers are located in parallel to the substrate, and their number possibly corresponds to the number of spraying steps.

Such differences can relate to molecular structures of the complexes. A more pronounced relief may be associated with the higher molecular weight of PAMPSA. In PANI-PAMPSA complex, PANI macromolecules, which are rigid in the conjugated emeraldine form, are surrounded by flexible-chain PAMPSA and formed coiled chains uniformly distributed on the substrate. Such complex presumably should have a double-strand structure [22,42]. PANI-PSSA has a fine nanorelief in spite of the similar flexible backbone of PSSA. However, the phenyl containing side chains bearing sulfonic groups sterically hinder free bending of the PSSA chain. At the same time, large height fluctuations and clearly defined dried-up droplets can be caused by the greater hydrophobicity of the PSSA.

In the case of rigid-chain t-PASA, PANI macromolecules are presumably located perpendicular to several macromolecules of the rigid-chain polyacid [22] and form less mobile rigid structures which can be arranged in parallel layers during spraying. The complex with semi-rigid PANI-i-PASA has a mixed structure [22] in which some of the fragments of PANI macromolecule units are surrounded by the polyacid chains, while others are linked by the polyacid with the neighboring ones. This may lead to the imperfect mutual packing of the macromolecules and, accordingly, to aggregation and sedimentation. Such an imperfect structure results in a slight deterioration in sensory properties.

Based on the morphology investigation and differences in the complexes’ structure, we suppose that PANI-t-PASA film with more uniform, self-organized morphology and layered structure after the treatment in CaCl_2_ can effectively detect ammonia at concentrations more than 25 ppm with high sensitivity and low response time.

### 3.4. Sensing Properties

**Figure 7:**
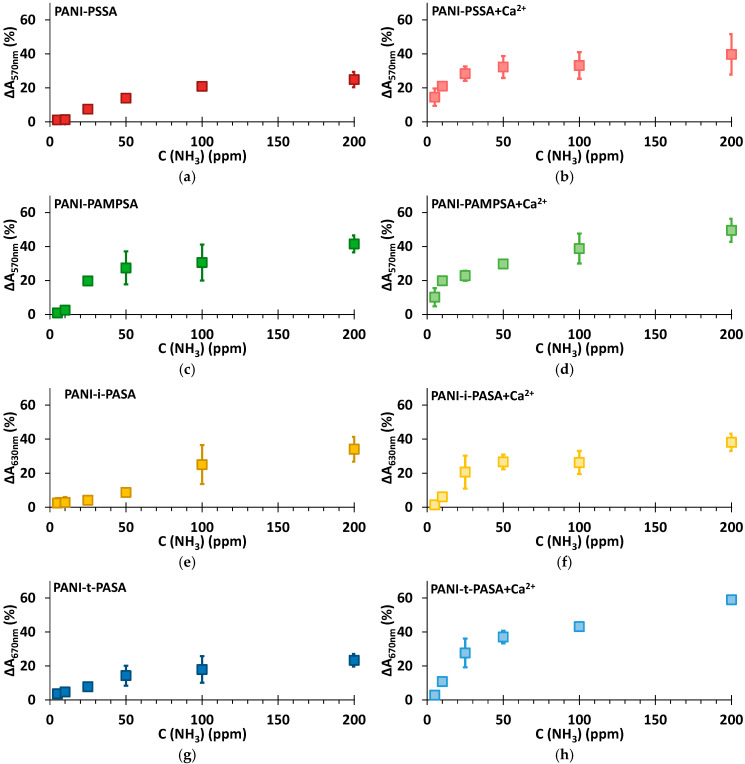
Dependence of response amplitude on the different concentrations of ammonia for the films of PANI complexes with PSSA (**a**), PSSA+Ca^2+^ (**b**), PAMPSA (**c**), PAMPSA+Ca^2+^ (**d**), i-PASA (**e**), i-PASA+Ca^2+^ (**f**), t-PASA (**g**) and t-PASA+Ca^2+^ (**h**).

From the dependences of the maximum response amplitude (ΔA) at the characteristic wavelengths on the concentration of ammonia in air (Figure 7), it is evident that PANI complexes with the flexible-chain polyacids (PSSA and PAMPSA) after the treatment in CaCl_2_ can detect ammonia at very low concentrations (5 and 10 ppm) and demonstrate high response amplitude at 25 ppm (Figure 7b,d). The untreated PANI-PAMPSA film demonstrates the highest response amplitude among all untreated films (Figure 7c). PANI complexes with the rigid-chain polyacids, especially t-PASA, after the treatment determine ammonia in the air with high response amplitude in the range of concentrations above 25 ppm (Figure 7h). The saturation of ammonia-sensing response for PANI-PSSA and PANI-i-PASA appears after 50 ppm (Figure 7b,f). In the case of PANI-PAMPSA and PANI-t-PASA the growth of ΔA continues up to higher ammonia concentrations (Figure 7d,h). Importantly, standard deviation of the determination is the lowest for PANI-t-PASA film (Figure 7h).

It should be noted that the films of PANI complexes are reusable 2–4 times, the reversibility decreasing at high ammonia concentration. These films are therefore preferably used as alarm detectors.

If we compare the sensory properties of Ca^2+^-treated spray-coated PANI–polyacid films with those of electrodeposited PANI–polyacid films [12] (Table 2), one can see the influence of method of films preparation. The differences may relate to specific features of formation of PANI films by electrodeposition and spray coating: in the first case, the polyacid content in the film is mostly determined by the electrosynthesis conditions, while in the second case the polyacid content is predetermined by the composition of the synthesis solution. At the same time, there are several general patterns. The response amplitudes are higher for all PANI films prepared by spray coating, but the response times (except in PANI-t-PASA) are longer. The response amplitude of PANI-PAMPSA at 50 ppm is higher for both methods of preparation. Diffusion coefficient of spray-coated PANI-t-PASA film is the highest [12]. Electrochemically prepared PANI-PSSA film demonstrated the worst sensing properties due to its lower doping level [12]. On the contrary, the spray-coated PANI-PSSA complex has a response amplitude similar to other PANI complexes. Just as in the case of electrochemically obtained PANI films, treatment by CaCl_2_ leads to a significant increase in the sensitivity, especially for PANI-t-PASA film.

Thus, the electrodeposition method results in the sensor films with shorter response time, but it requires conducting substrates and expensive electrochemical equipment. The spray coating allows us to obtain films with higher sensitivity to ammonia in air on various substrates, particularly on flexible ones.

## 4. Conclusions

In the present work, we have first performed a comparative study of the optical ammonia-sensing properties of water-dispersible PANI complexes chemically synthesized in the presence of polysulfonic acids of different structures and chain flexibility. The sensor films were obtained by a convenient and scalable method—spray coating. The range of ammonia vapor concentrations from 5 to 200 ppm was studied. It was shown that the as-coated films of PANI complexes with the flexible-chain polyacids have the highest response amplitude in the range of ammonia concentrations 5–25 ppm, but it is subject to bigger fluctuation under the influence of humidity.

To overcome the influence of humidity on the accuracy of ammonia determination, treatments of the films in aqueous solutions of NaCl, CaCl_2_ and BaCl_2_ were tested. All three variants of treatment produced no influence on the electron absorption spectra of all films. These treatments were chosen to solve two problems—(1) to remove excessive protons of the polyacid, which can partly neutralize ammonia molecules penetrated into the films thus reducing the optical response amplitude and, therefore, the accuracy of determination; (2) to reduce the solubility and swelling of the spay-coated films of PANI water-dispersible complexes, which induce fluctuations in their optical absorbance. It was shown that the treatment of all of the films of PANI complexes in 1 M NaCl solution effectively neutralizes the excessive protons but does not solve the problem of swelling. The treatment in 1 M CaCl_2_ solution for all of the PANI complexes results in a significant improvement in the response time, amplitude and reproducibility. The treatment in 1 M BaCl_2_ solution also improved the ammonia-sensing properties of all the films, but the response amplitudes in this case were lower in general than those after the CaCl_2_ treatment. FTIR spectroscopy showed that CaCl_2_ treatment results in cross-linking of sulfoacid groups from adjacent polyacid chains by Ca^2+^ ions. Thus, such treatment results both in the neutralization of excessive protons and significant reduction in the films’ swelling.

AFM and SEM studies of the films revealed no changes in their morphology after all of the three treatments. From the analysis of AFM cross-sectional profiles of the films and their ammonia-sensing properties, it was concluded that macro-relief (on the scale of 40 × 40 mm) induced by spray-coating procedure has a smaller influence on the ammonia-sensing properties than the intrinsic nano-relief predetermined by the peculiarities of molecular structure of the PANI complexes.

Among the films of the PANI complexes treated with CaCl_2_ the best sensory properties demonstrated PANI-t-PASA film at ammonia concentrations more than 50 ppm. The films of PANI complexes give reproducible results at reusing 2–4 times, the reversibility decreasing at high ammonia concentration. So, at this stage of the investigations, these films are preferably to be used as alarm detectors.

## Figures and Tables

**Table 1:** Values of the sensor response amplitude (ΔA), response time (t_r_) and diffusion coefficient (D) at 50 ppm of ammonia (smell detection limit [40]) of the spray-coated PANI–polyacid films.

	ΔA at 50 ppm, %	t_r_, s (50 ppm)	D, 10^−12^, cm^2^/s
PANI-PSSA	10.3	171	2.5
PANI-PSSA+Ca^2+^	22.5	66	7.9
PANI-PAMPSA	12.4	378	3.4
PANI-PAMPSA+Ca^2+^	25.8	206	17.4
PANI-i-PASA	7.6	216	3.1
PANI-i-PASA+Ca^2+^	21.8	115	9.2
PANI-t-PASA	17.7	142	25.2
PANI-t-PASA+Ca^2+^	40.3	104	55.8

**Table 2:** Comparison of the sensor response amplitude (ΔA) and response time (t_r_), at 50 ppm of ammonia of the spray-coated (this work) and the electrodeposited PANI–polyacid films [12] treated with 1 M CaCl_2_ solution.

	Spray Coating		Electrodeposition	
	ΔA, %	t_r_, s	ΔA, %	t_r_, s
PANI-PAMPSA	25.8	206	18.0	70
PANI-t-PASA	40.7	104	19.7	194

## Data Availability

Data are contained within the article and Supplementary Materials.

## Supplementary Materials

The following supporting information “Supplementary materials. Optical ammonia sensors based on spray-coated polyaniline complexes with polysulfonic acids” can be downloaded at https://www.mdpi.com/article/10.3390/s25113348/s1.
