# Supplementary material for: Optical Ammonia Sensors Based on Spray-Coated Polyaniline Complexes with Polysulfonic Acids
Source: Sensors (Basel). 2025 May 26;25(11):3348. doi: 10.3390/s25113348 (PMC12157768; doi:10.3390/s25113348)
Supplement: Supplementary file 1 [file sensors-25-03348-s001.zip › sensors-3618265-supplementary.pdf]

Supplementary materials

Optical ammonia sensors based on spray-coated polyaniline complexes with polysulfonic acids

O.L. Gribkova, V.A. Kabanova, E.I. Rodina, M.T. Teplonogova, L.I. Demina, A.A. Nekrasov

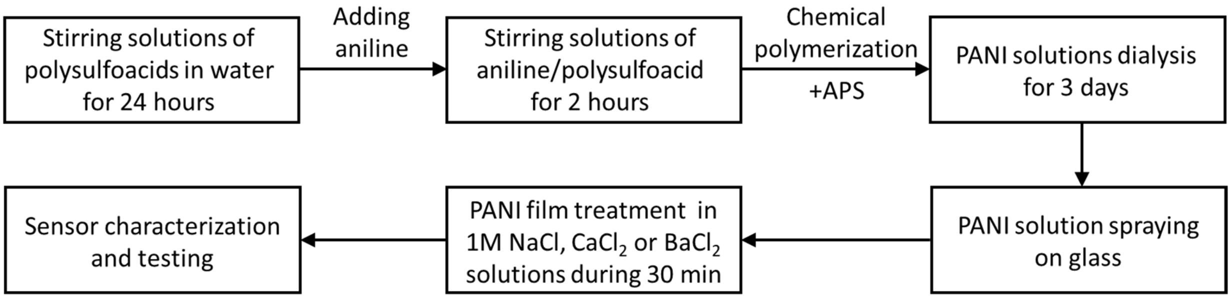

Figure S1. Flow chart of the experiments.

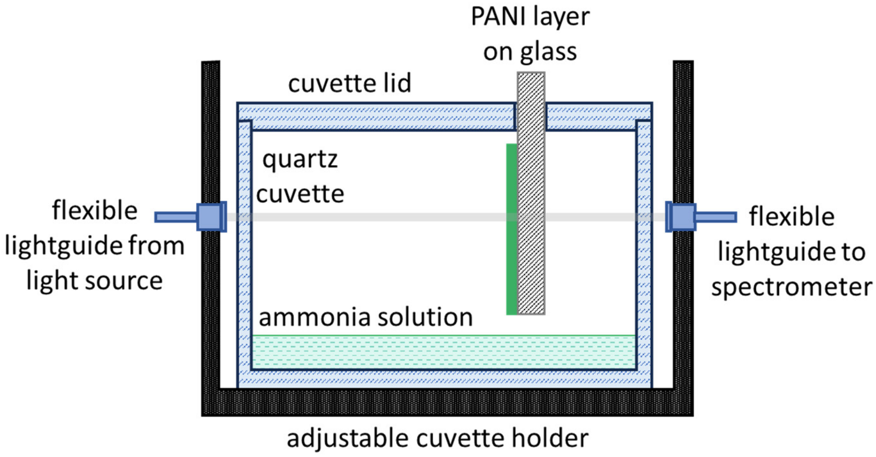

Figure S2. Scheme of ammonia sensing experiments.

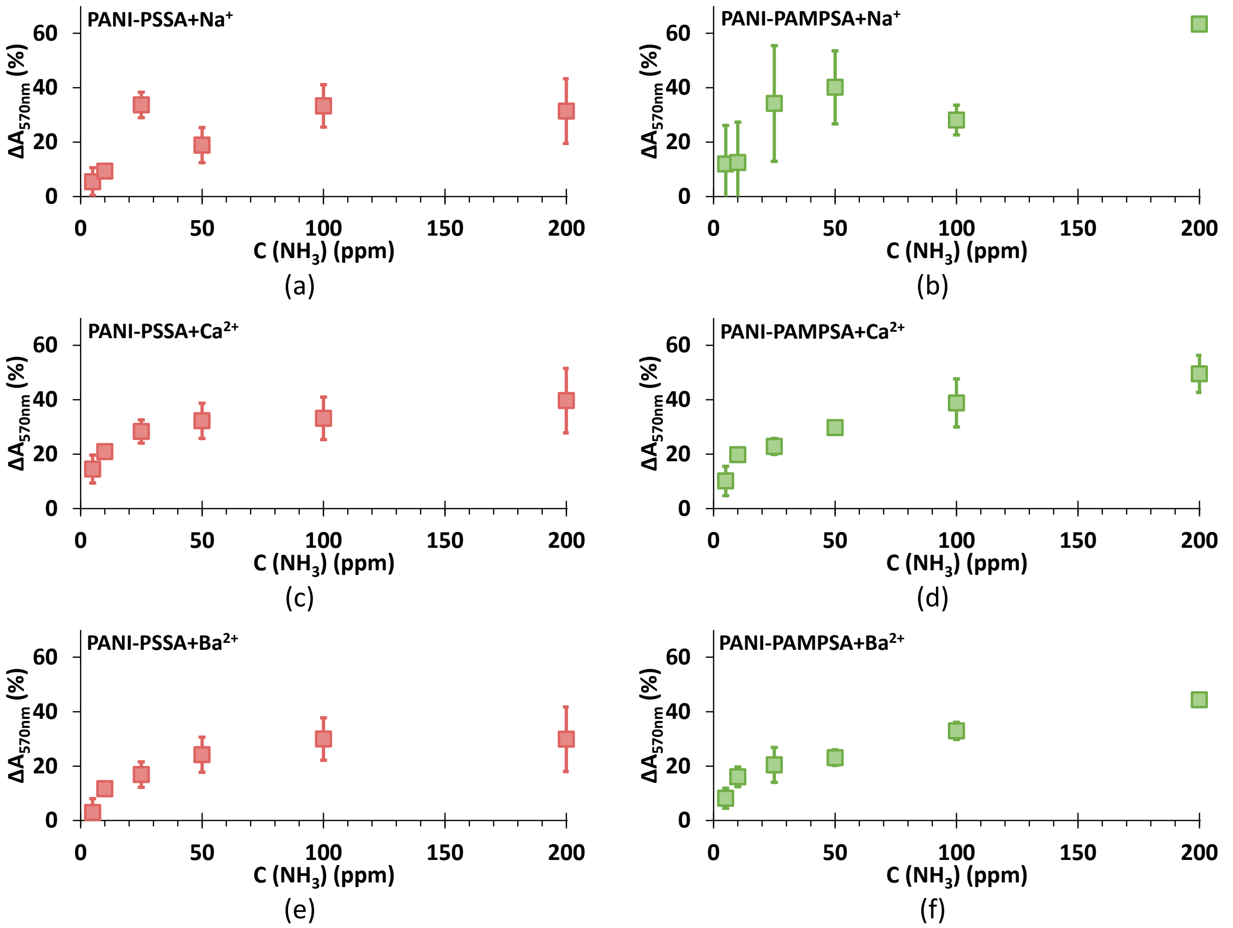

Figure S3. Dependence of response amplitude on the different concentrations of ammonia for the PANI-PSSA (a,c,e) and PANI-PAMPSA (b,d,f) films treated in 1M NaCl (a,b), 1M $CaCl_2$ (c,d) and 1M $BaCl_2$ (e,f).

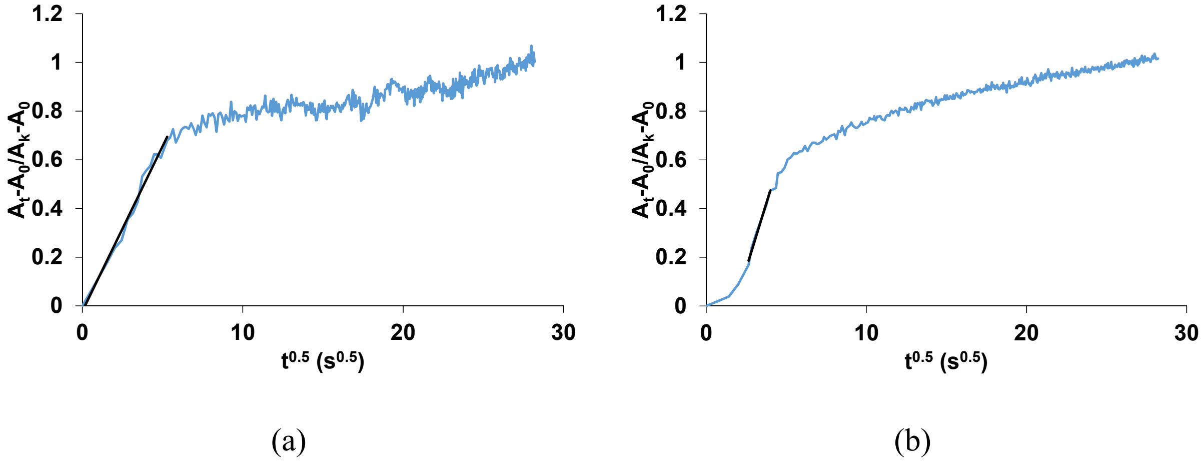

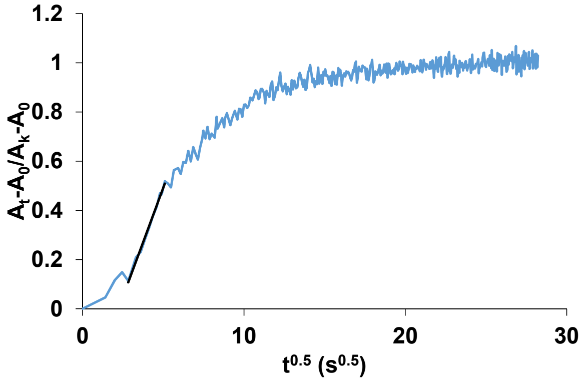

(c)

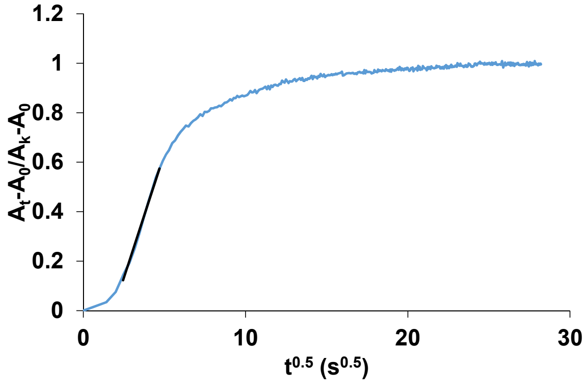

(d)

Figure S4. The dependences (A_t - A_0) / (A_k - A_0) on $t^{0.5}$ for $CaCl_2$ treated PANI-PSSA (a), PANI-PAMPSA (b), PANI-i-PASA (c) and PANI-t-PASA (d) exposed to 50 ppm of ammonia.

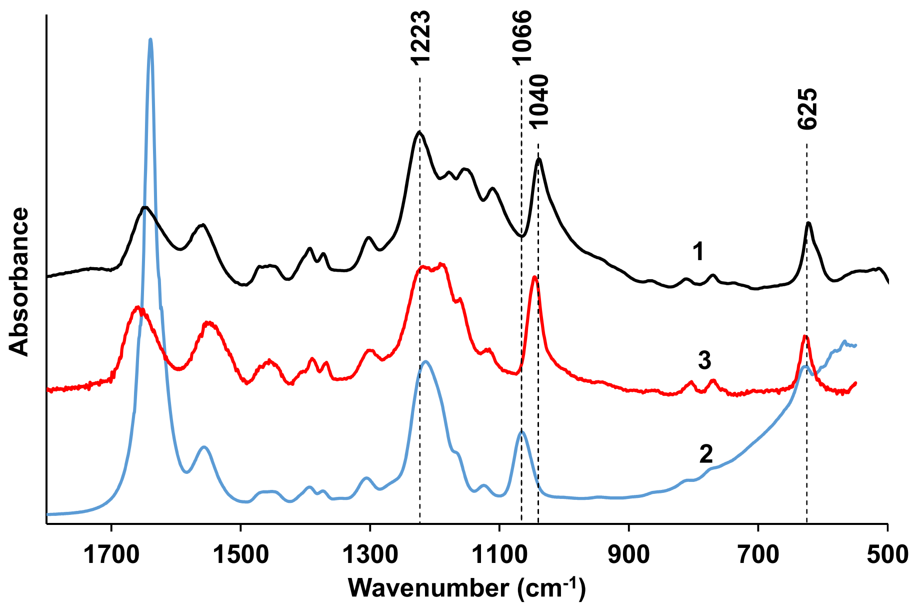

	ν_{as}(O=S=O)	ν_s(O=S=O)	ν(S-O)
PAMPSA	1223	1040	625
PAMPSA+Na^+	1218	1046	627
PAMPSA+Ca^{2+}	1214	1065	629

Figure S5. The FTIR spectra of PAMPSA film (1), treated with $CaCl_2$ (2) and NaCl (3).
